# Towards a better integrated stroke care: the development of integrated stroke care in the southern part of the Netherlands during the last 15 years (Special 10th Anniversary Edition paper)

**DOI:** 10.5334/ijic.744

**Published:** 2012-05-25

**Authors:** Ron Heijnen, Martien Limburg, Silvia Evers, George Beusmans, Trudy van der Weijden, Jos Schols

**Affiliations:** Vivre Group, Polvertorenstraat 6 6211 LX Maastricht, The Netherlands; Flevo Hospital, 1315 RA Almere, The Netherlands; Associate Professor at the Department of Health Organisation Policy and Economics, Maastricht University, 6200 MD Maastricht, The Netherlands; Associate Professor of General Practice, Maastricht University, 6200 MD Maastricht, The Netherlands; Professor of Implementation of Clinical Practice Guidelines, Department of General Practice, Maastricht University, 6200 MD Maastricht, The Netherlands; Professor of Nursing Home Medicine, Department of General Practice, Maastricht University, 6200 MD Maastricht, The Netherlands

**Keywords:** integrated care, stroke service, Netherlands

## Abstract

**Introduction:**

Stroke care is complex and often provided by various healthcare organisations. Integrated care solutions are needed to optimise stroke care. In this paper, we describe the development of integrated stroke care in the region of Maastricht during the last 15 years.

**Description of integrated care case:**

Located in the south of the Netherlands, the region of Maastricht developed integrated stroke care to serve a population of about 180,000 people. Integration was needed to improve the continuity, coordination and quality of stroke care. The development of integrated care in Maastricht was a phased process. The last phase emphasized early discharge from hospital and assessing the best individual rehabilitation track in a specialized nursing home setting.

**Discussion and lessons learned:**

The development and implementation of integrated stroke care in the region of Maastricht led to fewer days in hospital, more patients being directly admitted to the stroke unit and an earlier start of rehabilitation. The implementation of early discharge from the hospital and rehabilitation assessment in a nursing home led to some unforeseen problems and lessons learned.

## Introduction

There will be a marked increase in the number of stroke patients in Europe over the next decades [1]. By the year 2020, 250 per 100,000 inhabitants of the Netherlands will suffer from a stroke, often with subsequent permanent disabilities and handicaps as a consequence [2]. In terms of costs, stroke is among the most expensive diseases in the Netherlands with a total of 1.5 billion euros accounting for 2.2% of total annual health care costs [3]. Today, optimising stroke care in order to satisfy the demands for care, to enhance patient satisfaction and to be cost-effective is an important field of research worldwide [4–6].

Several randomised controlled trials have already shown that stroke care organised in hospital stroke units leads to a reduction in mortality, less dependency on care and a decrease in long-term institutionalised care [7]. During the last decade in the Netherlands, in addition to the development of hospital stroke units, there has been a trend towards the development of integrated regional stroke services, leading to more integrated care for stroke patients, to increase satisfaction among patients and caregivers, and last but not least, also leading to more cost-effective care [8]. This trend fitted also in an international trend towards integrated stroke care services [9, 10].

Nowadays, in accordance with the Helsingborg Declaration on European stroke strategies, stroke patients in the Netherlands are part of a continuous care chain from the moment the stroke occurs [11]. This continuous care chain is often embedded in stroke services which are an organisational model of integrated care for stroke patients. Integrated care can be seen as the result of multi-pronged efforts to promote a coherent set of methods and models on the funding, administrative, organizational, service delivery and clinical levels, designed to create connectivity, alignment and collaboration within and between the cure and care sectors, to enhance quality of care and quality of life, consumer satisfaction and system efficiency for patients with complex problems which cut across multiple services, providers and settings [12].

The last decade before the millennium was extremely important for the development and innovation of stroke care in the Netherlands. Changes were necessary because healthcare in the Netherlands, as well as in many other Western countries, was very fragmented. Stroke care lacked continuity, coordination, and communication often resulting in long hospital stays for stroke patients [13]. To improve this situation, a better coordination and cooperation between professional caregivers, often working for different care organisations, was strived for. The development of organised stroke care in the Netherlands, with specific stroke units and stroke services, started in the 1990s, stimulated by the Dutch Heart Association, which also released publications describing a step-by-step setup for stroke units and stroke services [14, 15]. National guidelines were developed, providing stroke care professionals with evidence-based recommendations for delivering optimum care [16, 17]. To further facilitate the implementation of stroke services, the Dutch Institute for Healthcare Improvement started a series of breakthrough projects for stroke care. In this nationwide effort, different regions were supported in implementing the integrated delivery of stroke service care [18].

Currently there are just over 100 general hospitals in the Netherlands, of which more than 70 participate in providing services for stroke patients. Although there are regional differences, most of these stroke services are collaborations between a general hospital, one or more nursing homes, a rehabilitation clinic and home care organisations. Most of the Dutch stroke services are affiliated with a knowledge network (“Kennisnetwerk CVA Nederland”) that strives towards implementing the goals set by the Helsingborg Declaration.

Besides providing chronic continuing care for somatic and psychogeriatric patients, nursing homes in the Netherlands have a specific geriatric rehabilitation function, whereas rehabilitation centres focus primarily on the rehabilitation of younger patients, who can cope with a more intense rehabilitation programme. Accordingly, Dutch nursing homes play a substantial role in integrated stroke care service, especially in the rehabilitation of elderly stroke patients. After hospital discharge, 32% of stroke patients return to their home, 9% are discharged to a rehabilitation centre and 31% are rehabilitated in a nursing home [8]. Dutch nursing homes employ their own nursing, paramedical and psychosocial staff and, in contrast to other Western countries, the medical treatment of nursing home patients is an officially recognized medical discipline, and nursing home physicians are specifically trained in this specialization. The nursing home sector in the Netherlands is mainly a non-profit sector, covered by a mandatory (national) insurance system for all citizens, the Exceptional Medical Expenses Act [19]. In 2001, with extra funding from the Exceptional Medical Expenses Act the stroke rehabilitation function of nursing homes was stimulated even further.

In the Netherlands, the development of integrated stroke care has stimulated the promotion of integrated care for other specialist services as well. Especially in the comprehensive care for diabetic patients and in the care for frail and disabled elderly, which often have complex care needs due to multiple co-morbidities, integrated care programs are being developed and implemented now nation wide [20, 21].

In comparison to the approaches in integrated stroke care in other countries, for instance the development of hyper-acute stroke units (HASUs) in Great Britain, the Dutch experience differs because of the unique abilities and positioning of Dutch nursing home care. HASUs are developed to enable more patients being treated with thrombolytic drugs by concentrating acute care for stroke patients in a few specialised centres, enabling admission and treatment of stroke patients 24 hours a day, 7 days a week. Patients admitted to a HASU will receive acute care for up to 72 hours after which they will be transferred to a stroke unit, also in the hospital setting, for further care and rehabilitation.

In the region of Maastricht, stroke patients are able to receive acute stroke care 24 hours a day, 7 days a week in the academic hospital and subsequently they are transferred to a nursing home for further assessment and rehabilitation.

This paper describes the development and changes over time of integrated stroke care in the south of the Netherlands, specifically in the region of Maastricht. In this development process, several phases can be distinguished which give insight into national and local factors that play a role in the integration of stroke care in the Netherlands. Parts of the changes in this development process were related to evaluations performed. In the last phase of this development, the stroke service underwent the last reformation, which will be described in detail.

## Towards integrated stroke service care in the Maastricht region

The Maastricht region has about 180,000 inhabitants; it is situated in the southernmost part of the Netherlands, close to the borders of Belgium and Germany. Maastricht has only one hospital, with 715 beds; this hospital provides standard medical care for the region, and also serves as an academic centre for about 1.1 million inhabitants. In 2010, 365 stroke patients were admitted to the academic hospital and received care within the stroke service Maastricht. The mean age of these stroke patients was 70 years (standard deviation 15).

Integrated care for stroke patients was not available in Maastricht before 1996. Stroke patients were treated by various individual health care providers without any coordination. In that period, the average hospital stay for stroke patients was 28 days, during which the patient received little rehabilitation therapy. In view of the importance of starting rehabilitation as soon as possible after stroke, this represented suboptimal care for recovery [22].

Since 1996, integrated stroke care services in Maastricht as well as in other regions started to thrive, due to the expected effectiveness of thrombolysis as a treatment for stroke [23]. This encouraged hospitals to enlarge their stroke unit capacity, enabling every stroke patient to be admitted directly to the stroke unit after the onset of stroke. In order to better coordinate the flow of stroke patients through the health care chain, the integrated care model for stroke patients was designed, later evolving into the stroke service Maastricht. The stroke service Maastricht involves collaboration between general practitioners, neurologists, rehabilitation specialists, nursing home physicians, psychologists, nursing staffs, district nursing, physiotherapists, speech therapists, occupational therapists and dieticians working for the academic hospital, the nursing home, the rehabilitation centre and in primary healthcare.

The total development process was characterised by four phases. During the first phase, which started in 1996, the focus was on achieving a better degree of cooperation between caregivers within the academic hospital itself. Next to this, caregivers of regular community care were involved. A protocol was developed in which the care process was described from the moment of stroke onset until discharge to the home situation and a collaborative training programme for visiting nurses, physiotherapists and general physicians was setup.

The goals set were:

1. The development of a care process in which as many stroke patients as possible could be admitted directly to the stroke unit of the academic hospital, as quickly as possible after the onset of stroke.
2. The duration of hospital stay for stroke patients should be as short as possible.
3. Community care, treatment and follow-up should start immediately after hospital discharge.

The second phase in the development of the stroke service Maastricht started in the year 2000. During this phase the emphasis was on the structured participation of the nursing homes in the region. The two nursing homes which in fact already participated in stroke service, but not in a structured way, were willing to reserve a total of 21 beds for older stroke patients who could be discharged from the academic hospital but couldn’t yet return home. The two nursing homes committed themselves to admitting stroke patients within 10 days after referral from hospital. To facilitate this fast transition, an agreement had to be reached with the central indicating commission for care (CIZ). In the Netherlands, the CIZ is charged with the assignment of care provided by nursing homes. Normally this means that patients need to be visited by a CIZ employee before being approved for rehabilitation in a nursing home. During such a visit the CIZ employee judges the clinical information from the hospital related to the functional status and prognosis of the patient. However, this may take a couple of days and lead to an unnecessary delay in the care process. Therefore it was agreed that stroke patients could be admitted directly to the nursing home, without waiting for a CIZ employee visit. The official indication could be provided at a later date.

This phase in the development of the stroke service Maastricht in fact ended with the results of a study conducted in Maastricht. This study compared stroke service care in Maastricht with care for stroke patients in a region without a stroke service. The results showed that 6 months after stroke 64% of the surviving patients in Maastricht could be discharged to their own homes, in comparison with 42% in the care as usual group, which was more fragmented and without any co-ordination [24].

In 2002 the third phase started. In this phase specific attention was paid to further improving the quality of stroke care by implementing all relevant recommendations from the most recent national guidelines on rehabilitation after stroke [17]. In addition, much work was done on improving communication and coordination between professional caregivers within and amongst organisations participating in the stroke service, by improving for instance, the quality of the transitional information. Agreement was also reached on which clinimetric tests should be used throughout the care chain. Clinimetric tests like the Assessment of Motor and Process Skills (AMPS), Barthel Index (BI) or the mini-mental state examination (MMSE) provide information on different functional levels. Using the same clinimetric tests at set times makes it possible to monitor a patient’s progress and improve communication between caregivers about the condition of the patient as well as about changes in this condition. Furthermore, care after discharge from the nursing home was improved as well, and structural education on the handling of psychological and behavioural effects of stroke was initiated for the patients and their caregivers.

**Figure 1:**
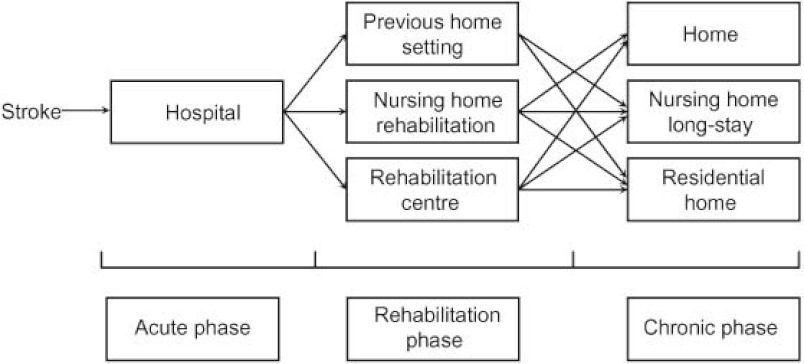
Stroke care model of the stroke service Maastricht in 2004.

After this phase, the development of organised stroke care in the region of Maastricht had resulted in a complete stroke service model, with the participation of an (academic) hospital, a large nursing home organization, a rehabilitation centre and a home care organisation and the model complied with the required model of stroke services in the Netherlands. Figure 1 depicts the model of Dutch stroke care in that time.

The fourth phase was developed after an evaluation of the integrated stroke service in 2004, which will be discussed below.

## Evaluation of the integrated stroke service Maastricht

Integrated stroke care in the region of Maastricht is constantly being monitored, not only by an implemented electronic registration system that enables the gathering of a set of important indicators on the quality of stroke care, but also by means of scientific studies which are regularly being carried out [24–26]. All evaluations are initiated by a steering committee consisting of representatives of all health organisations participating in the stroke service.

In 2004, the integrated stroke care service in Maastricht and its surrounding region was analysed scientifically for the first time, because the average hospital stay of a stroke patient still amounted to 12 days and not all stroke patients could be admitted directly to the hospital’s stroke unit. The study, carried out by Vos et al. [25] consisted of a process analysis, the identification of bottlenecks, the setting of goals and the selection as well as the implementation of coordination measures. The effects were measured by means of length of hospital stay and the number of patients admitted to non-specialised wards. Vos et al. identified the following barriers:

A first barrier involved the insufficient capacity of the stroke unit of the academic hospital. Because of this, 31% of stroke patients were not admitted directly to the stroke unit. A second barrier was presented by the time needed for initial diagnostic tests (such as: CT-scan, Echo Doppler of the carotid artery, Cardiac Echo, or a 24-ECG) and medical consultations to be carried out in the academic hospital. These diagnostic tests and consultations should have been carried out at the time of admission, but actually this took approximately three days. A third barrier was formed by the low frequency of the multidisciplinary meetings, which took place only once a week. The multidisciplinary meetings are meant to evaluate the triage process and to determine the further rehabilitation track for each individual patient. The low frequency of the meetings caused an increase in the length of hospital stay for patients who otherwise could have been discharged home earlier. A final barrier was formed by the waiting times for admission to the rehabilitation clinic and the nursing homes. All these barriers resulted in an average hospital stay of 12 days, of which on average 3 were superfluous from a medical perspective.

The identification of these barriers mandated a further redesign of the integrated stroke service Maastricht. This can be seen as the fourth phase in the development of the stroke service. Even more than in the past, the emphasis in this phase was laid on faster discharge from the academic hospital by better coordination and planning of initial diagnostic tests and consultations.

Apart from this, the multidisciplinary assessment and its related multidisciplinary meetings, to determine the best rehabilitation track (triage phase), which originally took place in the hospital, were transferred to the nursing home. In addition, the existing protocol for the rehabilitation of stroke patients in the nursing home had to be extended to incorporate an initial multidisciplinary assessment.

Because patients would be discharged much faster from hospital in the adapted model, the flow of patients to the nursing home was expected to increase, and therefore more nursing home beds were needed for assessment and rehabilitation. Accordingly, nursing home management decided to enlarge the nursing home stroke ward from 21 to 30 beds. Moreover, all 30 stroke beds were positioned in a single nursing home ward.

To assure that this nursing home stroke ward was able to receive new stroke patients at all times, the ward’s patient outflow had to be guaranteed. Therefore, stroke patients who had finished their rehabilitation in the nursing home but could not be discharged home were given priority in finding a permanent bed for continuing long-term care in a residential or nursing home ward of the participating nursing home organisation.

## Description of the redesigned integrated stroke service Maastricht

The redesigned integrated stroke service Maastricht involves a critical care pathway for stroke patients admitted to the academic hospital. In this redesigned care pathway every stroke patient is admitted directly to the hospital stroke unit. Most are referred by general practitioners and brought to the emergency ward of the hospital by ambulance, but some come on their own initiative without first consulting a general practitioner. In the emergency ward acute diagnostic tests take place. In cases of confirmed stroke, the patient will be admitted to the stroke unit of the academic hospital, where further diagnosis and treatment, including thrombolysis if indicated, are performed.

**Figure 2:**
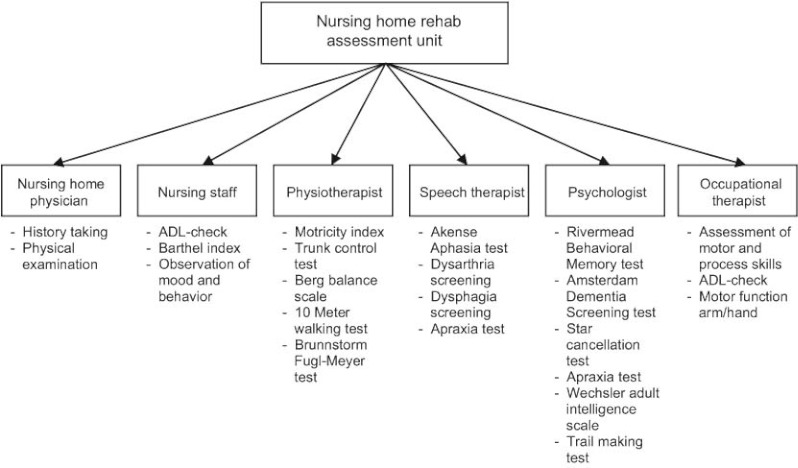
Content of assessment at the nursing home assessment unit.

Subsequently, the redesigned care model consists of a strict discharge regime for all stroke beds from the neurology ward of the academic hospital. All necessary tests and treatment in the hospital should be performed within 5 days after admission. Thereafter, in principle, all patients, regardless of their age, will be discharged to the stroke ward of the nursing home, where a comprehensive assessment takes place (Figure 2). Only patients who can be discharged home within 5 days after admission and those who are medically unstable will not be transferred from the hospital to the nursing home within 5 days.

The nursing home physician examines each patient immediately on arrival in the nursing home and initiates the assessment program. In this program a multidisciplinary team consisting of a psychologist, physiotherapist, occupational therapist, speech therapist and trained nurses examine the patient, performing a structured assessment protocol. Following this assessment, the team will meet within five days of the patient’s admission to make recommendations for a rehabilitation program specifically tailored to the patient. Their advice will be based on admission and discharge criteria formulated by the various care providers participating in the stroke service. There is a structured possibility for the nursing home physician to consult a rehabilitation physician if needed. After the multidisciplinary meeting, the patient and his family will be informed about the proposed rehabilitation track; if they approve this track can be started.

There are three options for a rehabilitation track after the assessment in the nursing home.

1. Rehabilitation at home with home care and outpatient treatment provided by therapists from primary healthcare or day care rehabilitation in a hospital or nursing home.
  In cases of fast functional recovery after stroke with the availability of adequate informal care and a safe environment at home.
2. Inpatient rehabilitation in a nursing home.
  In cases where stroke patients need a prolonged rehabilitation trajectory of a lower intensity.
3. Inpatient rehabilitation in a rehabilitation centre.
  In cases where the patient is in need of high-intensity rehabilitation, and/or reintegration into regular work activities.

**Figure 3:**
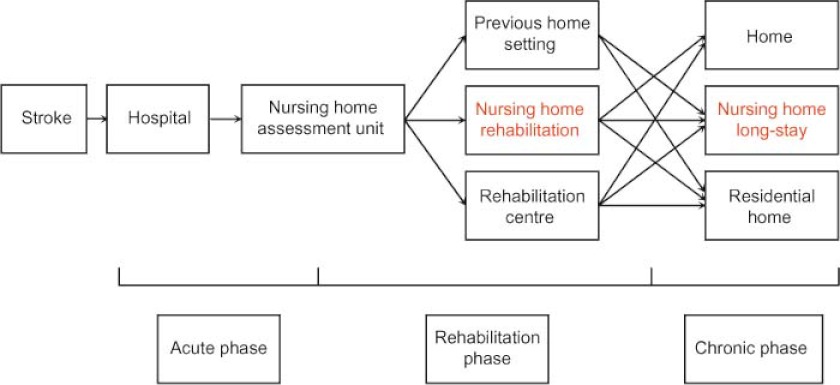
The redesigned integrated stroke service Maastricht.

The redesigned integrated stroke service Maastricht is displayed in Figure 3.

This new care model for stroke patients was implemented in January 2006. During the first four months following implementation, data on the duration of hospital stay and admission directly to the stroke unit were collected for all stroke patients admitted to the academic hospital. The data showed that the duration of hospital stay had decreased to an average of 7.3 days and that the percentage of stroke patients who could not be admitted directly to the stroke unit had decreased to 2% [25]. Although this study showed a decrease in hospital stay and in the number of patients who could not be admitted directly to the stroke unit, the study did not take into account functional outcomes at patient level, quality of life and satisfaction with care.

Accordingly, the question remained whether in the new care model hospital stay was decreased without having a negative effect on other outcomes, such as the patient’s functional level, quality of life or satisfaction with care. To answer these questions and to depict the total costs of this stroke care model, a cost-effectiveness study is presently investigating the cost-effectiveness of the new care model [26]. This cost-effectiveness study consists of an effect evaluation, an economic evaluation and a process evaluation. The design of this study involves a non-randomised comparative trail for two groups. The participants are followed for six months from the time of stroke. The mean outcome measures of the effect evaluation are quality of life and daily functioning. In addition, an economic evaluation will be performed from a societal perspective. A process evaluation will be carried out to evaluate the feasibility of early discharge and assessment in a nursing home, as well as the experiences and opinions of patients and professionals. The first results of this study can be expected as early as July 2012.

## Lessons learned

Before the implementation of the last redesigned stroke service model could begin, stroke care professionals of different backgrounds worked together to define appropriate adaptations of the initial stroke care protocol. In the new stroke care protocol, admission and discharge criteria were formulated for every link in the stroke care chain and agreement was reached on what tests should be done by which professional at what point of time. Furthermore, the information needed for the effective transition of patients throughout the care chain was evaluated and adjusted. Despite this careful preparation of the new stroke care protocol, the implementation brought forward some unforeseen problems. These problems were expressed in contacts with the different stake holders of the stroke service including patients and health care professionals.

First, the relative unfamiliarity of patients in the region of Maastricht and surroundings with the possibilities of assessment and rehabilitation in a nursing home caused a problem. Experiences with the first patients showed that in general patients didn’t associate a nursing home with a quick discharge to their own home, but with a long or even permanent residency.

Therefore, some patients initially refused to their admission to the nursing home, but the hospital staff almost always succeeded in convincing them that this was the fastest way of starting rehabilitation. To change the patient’s views and to actually show the possibilities of rehabilitation in a nursing home, a better way of providing information to patients and their caregivers was arranged. Verbal information given by the hospital nursing staff was supplemented by a DVD which showed the different rehabilitation tracks in detail. This DVD was given to every stroke patient who was admitted to the academic hospital and their primary caretaker.

Second, for the healthcare professionals working in the new stroke care model, early discharge from the academic hospital in combination with assessment in the nursing home implied a shift in tasks. Some professionals in the hospital lost their function in the assessment of stroke patients, when that was adopted by the professionals in the nursing home. As an earlier study by van Raak showed, this can be perceived as a threat by some of the hospital professionals [27]. For instance, the rehabilitation specialists in the hospital, who lost their coordinating role in the triage process, had some difficulty in adapting to this shift, particularly in relation to their decision-making power. In the old stroke care model, the rehabilitation specialist coordinated the decision on the type of rehabilitation track the stroke patient should follow after hospital discharge. In the new care model, the triage function of the academic hospital and the related multidisciplinary meetings were transferred to the nursing home team, supervised by the nursing home physician, with only a consulting role for the rehabilitation specialist. In practice this occasionally caused a difference in opinion, but subsequent adequate communication always led to a patient friendly solution.

Third, in the new model, the patient’s transfer from the hospital to the nursing home is coordinated by the “discharge office of the academic hospital”. A staff member of this office visits the patients prior to discharge, informs them of the rehabilitation track to be followed and arranges transfer, if needed. This function is vital for maintaining an adequate and continuous patient flow. But because the two employees consigned to this task initially hadn’t coordinated their working hours, transfers could not always be planned in time. A better coordination of working hours solved this problem.

Fourth, another unforeseen problem was that the transport of the patients from the academic hospital to the nursing home hadn’t been discussed with the ambulance service before the start of the new care model. Because the ambulance service maintained previously made arrangements, patients often arrived at the nursing home too late in the day to start the assessment on arrival. This problem was solved by making good additional arrangements with the ambulance service.

Fifth, labelling extra beds for stroke patient assessment in the nursing home meant that the hospital became less ‘vulnerable’ to fluctuations in patient flows, in contrast to the nursing home, which needed extra capacity to cope with patient flow fluctuations. In times of low demands for stroke beds, the hospital was able to fill its beds with other neurology patients whereas the nursing home could not. In order to fulfil their part in the stroke service, the management of the nursing home was willing to keep their designated beds, even when unoccupied, and bear subsequent financial losses. Nowadays these problems are solved by additional reimbursement for nursing homes.

It can be concluded that by gradually altering the structure of the conventional stroke service model we have created a new care model that, based on evidence elsewhere, we expect to shorten the duration of hospital stay and lead to lower costs. Moreover this new model may have positive effects on patients’ functional outcomes, quality of life and satisfaction with care.

Currently we are investigating the added value of this new model. If the expected positive effects are established, the model might also be tested in integrated care models related to other chronic diseases. In this respect we can think of patients with chronic heart failure or of elderly patients who often stay hospitalised unnecessarily long because of their multimorbidity and complex care problems.

Optimizing integrated stroke care means knowing and using the abilities of different healthcare providers for a common purpose. In the Netherlands, nursing homes with their unique ability to equally participate in the rehabilitation of mostly elderly patients, take away the pressure from acute care providers, not only as part of a stroke service but also as part of other integrated care models.

## Reviewers

**Wilco Achterberg**, Professor of institutional care and elderly care medicine, Department of public health and primary care, Leiden University Medical Center, The Netherlands

**Caroline van Heugten**, PhD, Professor, Department of Neuropsychology and Psychopharmacology, Faculty of Psychology and Neuroscience; School for Mental Health and Neuroscience, Department of Psychiatry and Neuropsychology, Faculty of Health, Medicine and Life Sciences, Maastricht University, The Netherlands

**Grace Warner**, PhD, Dalhousie University, Halifax, Nova Scotia, Canada
